# Identification of long noncoding RNAs downregulated specifically in ovarian high‐grade serous carcinoma

**DOI:** 10.1002/rmb2.12572

**Published:** 2024-04-03

**Authors:** Maki Hayashi‐Okada, Shun Sato, Kengo Nakashima, Takahiro Sakai, Tetsuro Tamehisa, Takuya Kajimura, Isao Tamura, Kotaro Sueoka, Norihiro Sugino

**Affiliations:** ^1^ Department of Obstetrics and Gynecology Yamaguchi University Graduate School of Medicine Ube Japan

**Keywords:** ADAMTS9‐AS2, CBR3‐AS1, HGSC, lncRNA, NBR2

## Abstract

**Purpose:**

To investigate whether long noncoding RNAs (lncRNAs) are involved in the development or malignant behavior of ovarian high‐grade serous carcinoma (HGSC), we attempted to identify lncRNAs specific to HGSC.

**Methods:**

Total RNAs were isolated from HGSC, normal ovarian, and fallopian tube tissue samples and were subjected to a PCR array that can analyze 84 cancer‐associated lncRNAs. The lncRNAs that were upregulated and downregulated in HGSC in comparison to multiple samples of normal ovary and fallopian tube were validated by real‐time RT‐PCR. To infer the function, ovarian cancer cell lines that overexpress the identified lncRNAs were established, and the activation of cell proliferation, migration, and invasion was analyzed.

**Results:**

Eleven lncRNAs (ACTA2‐AS1, ADAMTS9‐AS2, CBR3‐AS1, HAND2‐AS1, IPW, LINC00312, LINC00887, MEG3, NBR2, TSIX, and XIST) were downregulated in HGSC samples. We established the cell lines that overexpress ADAMTS9‐AS2, CBR3‐AS1, or NBR2. In cell lines overexpressing ADAMTS9‐AS2, cell proliferation was suppressed, but migration and invasion were promoted. In cell lines overexpressing CBR3‐AS1 or NBR2, cell migration tended to be promoted, although cell proliferation and invasion were unchanged.

**Conclusion:**

We identified eleven lncRNAs that were specifically downregulated in HGSC. Of these, CBR3‐AS1, NBR2, and ADAMTS9‐AS2 had unique functions in the malignant behaviors of HGSC.

## 1 INTRODUCTION

Ovarian cancer is a gynecological cancer with a poor prognosis. There are various histological subtypes of ovarian cancer, with the most common being high‐grade serous carcinoma (HGSC), low‐grade serous carcinoma, mucinous carcinoma, endometrioid carcinoma, and clear cell carcinoma. Each subtype has its own distinct tumor origin, genes involved in tumorigenesis, and clinical presentation.^1^ Among ovarian cancers, HGSC is the most common histological type, accounting for 70% of all ovarian cancers, and mostly originates from the fallopian tube epithelium. It is one of the most rapidly growing human malignancies and most patients are diagnosed at an advanced stage. Therefore, it has a poor 5‐year survival rate of 9%–34%.^2^ Thus, it is essential to elucidate the molecular mechanisms involved in the development and malignant behaviors of HGSC.

The Cancer Genome Atlas (TCGA) project has elucidated several causative genes in HGSC, including TP53 mutations, but few functionally important gene mutations other than TP53 have been found. In contrast, a wide range of regions with chromosomal copy number abnormalities have been found, suggesting that the amplification of oncogenes and deletion of tumor suppressor genes contribute to the progression of HGSC.^3^ Whole‐genome studies have shown that the coding genes account for less than 2% of the genome, and it has become apparent that aberrations within the noncoding genome drive important cancer phenotypes.^4^ In HGSC, not only genetic abnormalities but also noncoding RNA (ncRNA) expression abnormalities are thought to occur at a high frequency and may cause cancer. Long noncoding RNAs (lncRNAs) are defined as transcripts of more than 200 nucleotides that are not translated into proteins, and many of them are uniquely expressed in differentiated tissues or specific cancer types.^5^ Single nucleotide polymorphisms (SNPs), copy number alterations, or mutations within the noncoding genome can dramatically alter lncRNA transcription. The prostate cancer risk‐associated SNP rs11672691 upregulates the lncRNA isoform PCAT19‐long, which promotes cell proliferation, tumor growth, and metastasis.^6^ Based on genome‐wide copy number variations, LOC101927151, LINC00861, and LEMD1‐AS1 were identified as prognosis‐associated lncRNAs in ovarian cancer.^7^ Many lncRNAs have been found to be specifically upregulated or downregulated in various cancers. In addition, the detailed mechanisms of action of some of them are now being elucidated. In ovarian cancer, several lncRNAs including H19, HOTAIR, MALAT1, and PVT1, have been reported to be involved in malignancy.^8^, ^9^, ^10^ However, there are few reports on these lncRNAs in HGSC. Nicholas et al.^11^ showed that 1943 of 16 325 lncRNA sequences were differentially expressed in HGSC in comparison to normal fallopian tube tissue, and identified three lncRNAs (MON2‐AS1 (AC079035.1), LINC00399, and AC002115.1) associated with survival in HGSC. Recently, in our laboratory, four aberrantly expressed lncRNAs (MEG3, POU5F1P5, ADAMTS9‐AS2, and XIST) were identified in ovarian cancer cell lines and primary ovarian surface epithelial cells and subsequently validated using multiple HGSCs and ovarian tissues.^12^

In this study, to further identify the lncRNAs specific to HGSC, we screened and validated lncRNAs in multiple HGSC tissues and normal ovarian and fallopian tube tissues, which are the possible origins of HGSC, and identified new lncRNAs specific to HGSC. In addition, to investigate whether the identified lncRNAs are involved in the development and malignancy of HGSCs, ovarian cancer cell lines in which the expressions of some of these lncRNAs was altered were established, and subjected to an in vitro functional analysis, resulting in the identification of some lncRNAs involved in the malignant behaviors of HGSC.

## 2 METHODS

### 2.1 Patient tissue samples and cell culture

Twenty‐two HGSC tissue samples were provided by Shimane University Hospital (details of clinical data are shown in Table S1). HGSC was diagnosed based on histological findings. Immunohistochemical staining with p53 or WT‐1 is not routinely performed for the pathological diagnosis of ovarian cancers in clinical practice.

Ten normal ovarian tissues and ten normal fallopian tube tissues were obtained from patients who underwent salpingo‐oophorectomy and salpingectomy at Yamaguchi University Hospital. The clinical and pathological diagnoses are shown in Tables S2 and S3. No intraepithelial carcinoma components were found in the fallopian tube tissue samples. None of the family histories of the patients from whom normal ovary and fallopian tube tissue samples were obtained suggested the presence of hereditary breast and ovarian cancer (HBOC). After the specimens were extracted, pieces of tissue were immediately immersed in liquid nitrogen and stored at −80°C. The study was approved by the Ethical Review Committee for Medical Research of our hospital for the collection of tissue specimens (No. H27‐216). Written informed consent was obtained from all patients.

In this study, two human ovarian cancer cell lines, CaOV3 and OVCAR3, were used as the HGSC cell models. CaOV3 cells were purchased from American Type Culture Collection (ATCC, Manassas, VA, USA) and cultured in Dulbecco's modified Eagle's medium (Wako, Osaka, Japan) supplemented with 10% fetal bovine serum (FBS). OVCAR3 cells were provided by The Cell Resource Center for Biomedical Research, Institute of Development, Aging and Cancer, Tohoku University, and cultured in RPMI‐1640 (Wako) supplemented with 20% FBS. All cell lines were tested for mycoplasma.

### 2.2 RNA isolation

Total RNA was isolated from HGSC and normal ovarian tissue samples using the AllPrep DNA/RNA Mini Kit (Qiagen, Hilden, Germany) according to the manufacturer's instructions. Total RNA was isolated from normal fallopian tube tissue samples using ISOGEN reagent (Nippon Gene, Tokyo, Japan), followed by chloroform extraction and 2‐propanol precipitation.

### 2.3 PCR array

Six HGSC samples and three normal ovarian and fallopian tube samples were randomly selected. cDNA was synthesized using an RT2 First Strand Kit (Qiagen), according to the manufacturer's instructions. The cDNA was analyzed using an RT2 lncRNA PCR Array Human Cancer PathwayFinder (Qiagen, GeneGlobe ID LAHS‐002Z), which can analyze 84 types of lncRNAs reported to be related to various cancers.^12^ Relative expression levels were calculated using the ΔΔCq method with ACTB as a reference gene. Significant changes in the expression levels of the selected candidate lncRNAs were defined as at least twofold upregulation or downregulation of the expression in HGSC in comparison to normal ovary and fallopian tube tissue samples (*p* < 0.05).

### 2.4 Real‐time reverse transcription polymerase chain reaction (RT‐PCR)

Twenty‐two HGSC samples and ten cases each of normal ovarian and fallopian tube tissue samples were used for real‐time RT‐PCR. cDNA was synthesized from 1 μg of total RNA using random hexamers with a Quantitect Reverse Transcription Kit (Qiagen), as previously reported.^13^ Real‐time RT‐PCR was performed using Luna Universal qPCR Master Mix (NEB, Ipswich, MA, USA) and the primer sets listed in Table 1, under the cycling conditions (45 cycles of 95°C for 15 s and 60°C for 30 s with an initial step of 95°C for 60 s). Relative expression levels were calculated using the ΔΔCq method, with GAPDH as a reference gene.

**TABLE 1:** PCR primers used for RT‐PCR in this study.

Primer name	Usage	Forward	Reverse
ACTA2‐AS1	RT‐PCR	GCTGTTCCAAGAATGTTGCTTTACT	ATAATTGAGGCAGAAATCACCAACC
ADAMTS9‐AS2	RT‐PCR	GAAGGATGTGCTTGGGAACTTTAAG	CTTTCCTTATCCTCAGCTTCTCACT
CBR3‐AS1	RT‐PCR	GCAGTAAGTGGTGTAAATTCCCTTT	TGCTAATAAAGGGCTACACAACTCA
HAND2‐AS1	RT‐PCR	CAAAGAACACGAGATGCCATTTCT	GAAAGAGGAGAAGAGGAAGAAGGAG
IPW	RT‐PCR	CTGGGAGTGAATGTTATCAGCAAAT	ACCAACTCAACAAATCCACCTCTTT
LINC00312	RT‐PCR	CTTCTTAATCTGGCTGTTGTTGTGT	CTTAGTACCTGGGCTCTGTTTAACT
LINC00152 (CYTOR)	RT‐PCR	AATATGACAGACACCGAAAATCACG	CATGACCAAAATATCACAGGCAGAC
LINC00887	RT‐PCR	GTGCCTGGTTATATTACTGGATCCT	GTGACTTCAGACAATTTCAGCCTC
MEG3	RT‐PCR	GCCATCACCTGGATGCCTAC	AGTCTCTGGGTCCAGCCTGT
NBR2	RT‐PCR	CCATAAAGTGCCTGCCCTCTAG	GATTGGGACCTCTTCTTACGACTG
TSIX	RT‐PCR	TCATTCTCTTTCTTTTGGAGGCAAC	AGAGCAAGACTTAGCAGGGAATAAA
XIST	RT‐PCR	ACGCTGCATGTGTCCTTAGTAG	TTGGAGCCTCTTATAGCTGTTTG
GAPDH	RT‐PCR	AGGTGAAGGTCGGAGTCA	GGTCATTGATGGCAACAA
ADAMTS9‐AS2	Construction^a^	AAACTTGACGTACACACGCA	TTCTGTTTTTATAATGTACA
CBR3‐AS1	Construction^a^	AGCCGCGGTGTGAGGGAGCG	ATCATAAAATGTTGAGTATC
NBR2	Construction^a^	GGATGACGTAAAAGGAAAGA	TTCATCAGAGGCTGGACTCT

^a^ Construction primers were created by adding the forward (ATTAAGGATCCTCGAGTAGGCCACC) and reverse (CCTGCGGTCGCGGCCGC) sequences for infusing the primer into the vector.

### 2.5 Overexpression of the lncRNAs in HGSC cell lines

Since the expression levels of the lncRNAs detected in this study were remarkably low in the cell lines (CaOV3 and OVCAR3), overexpression experiments were used to explore their functions. To induce the overexpression of three lncRNAs (ADAMTS9‐AS2, CBR3‐AS1, and NBR2), we first constructed their expression vectors. The full‐length cDNA of each lncRNA cDNA was amplified by RT‐PCR using cDNA of normal ovary tissue as a template, and PrimeSTAR GXL DNA polymerase (Takara, Kyoto, Japan) and the primer sets listed in Table 1, under the cycling conditions for ADAMTS9‐AS2 and NBR2 (35 cycles of 98°C for 10 s, 55°C for 15 s, and 68°C for 3 min), for CBR3‐AS1 (25 cycles of 98°C for 10 s, 60°C for 15 s, and 68°C for 3 min).

The amplified lncRNA fragments were inserted into the multicloning site of the pMXs‐IRES‐Bsd vector (Cell Biolabs, San Diego, CA, USA) using the In‐Fusion HD Cloning Kit (Takara). Sequencing of the constructed vectors confirmed that the target lncRNA had been inserted into the correct sequence. The constructed lncRNA expression vectors and control vector (nontreated pMXs‐IRES‐Bsd vector) were transfected into HEK293T cells (Takara) using Lipofectamine 3000 (Invitrogen, Carlsbad, CA, USA) to produce the retroviral vector packaged with the expression vectors. The retroviral vectors were added to CaOV3 and OVCAR3 cells plated at 50% confluence in 24‐well plates. Stable cell lines were established by sorting with 4 μg/mL blasticidin S for CaOV3 and with 2 μg/mL blasticidin S for OVCAR3 for a month.

### 2.6 Cell proliferation assay

The stable cell lines overexpressing lncRNAs and the control cell lines were plated at approximately 2 × 10^5^ cells per well in 6‐well plates. Every 24 h, a single‐cell suspension was prepared for each line by trypsinization and counted using a Vi‐CELL XR cell counter (Beckman Coulter, Tokyo, Japan). Each experiment was performed in triplicate.

### 2.7 Wound healing assay

Cells were cultured in a six‐well plate until they reached confluence. Linear scratch wounds were created in the center of each well with a 200‐μL sterile pipette tip. To evaluate wound healing, images were obtained at 0 and 72 h after the creation of scratch wounds, at the same fields under the microscope at 40× magnification, and the areas between the wounds were measured using the ImageJ software program. Wound healing activity was calculated by dividing the migration distance at 72 h by the original distance at 0 h. Each experiment was performed in triplicate and mean values were obtained from three independent experiments.

### 2.8 Migration and invasion assays

Migration and invasion assays were performed using a BioCoat Matrigel Invasion Chamber (Corning Life Sciences, Tewksbury, MA, USA) according to the manufacturer's protocol as previously reported.^14^ Cells were trypsinized into single cells and seeded at 1 × 10^5^ cells in serum‐free medium into the Matrigel‐coated and uncoated upper insert chambers of the transwell system. The lower chamber was filled with a medium containing 10% FBS as a chemoattractant. After 24 h in culture for CaOV3 and 48 h in culture for OVCAR3, the upper surfaces of the insert chamber membranes were wiped with cotton swabs to completely remove the remaining cells. The membranes of the insert chambers were fixed and stained using Diff‐Quick (Sysmex, Kobe, Japan). The stained cells were counted at 100× magnification in three randomized field views and the mean number was calculated. The migration activity was evaluated based on the number of cells that migrated to the lower surfaces of the uncoated membranes. Invasion activity was calculated as the number of cells that migrated to the lower surfaces of the Matrigel‐coated membranes divided by the number of cells that migrated to the lower surfaces of the uncoated membranes. Each experiment was performed in triplicate, and mean values were obtained from three independent experiments.

### 2.9 Statistical analysis

The significance of differences between two groups was analyzed using Student's *t*‐test. The significance of differences among three groups was analyzed using Kruskal–Wallis and Wilcoxon tests. *p* Values of <0.05 were considered to indicate statistical significance in comparisons between two groups, while *p* < values of 0.0167 were considered to indicate statistical significance in comparisons among three groups. All statistical analyses were performed using the R software program (Ver 4.1.0).

## 3 RESULTS

### 3.1 Identification of lncRNAs expressed specifically in HGSC

To identify lncRNAs expressed specifically in HGSC, the expression levels of lncRNAs in HGSC tissues were compared to those in normal ovarian tissues and normal fallopian tube tissues using a PCR array which can comprehensively analyze the expression of 84 lncRNAs known to be associated with various ctypes of cancer. Of the 84 lncRNAs analyzed, one upregulated lncRNA (LINC00152) and 11 downregulated lncRNAs (ACTA2‐AS1, ADAMTS9‐AS2, CBR3‐AS1, HAND2‐AS1, IPW, LINC00312, LINC00887, MEG3, NBR2, TSIX, and XIST) were identified as candidates for HGSC‐specific lncRNAs (Table 2).

**TABLE 2:** lncRNAs expressed specifically in HGSC by PCR array.

Symbol	Comparing to fallopian tubes			Comparing to ovaries		
	Fold change	95% CI	*p*‐Value	Fold change	95% CI	*p*‐Value
*Upregulated in HGSC*						
LINC00152	5.6086	(2.47, 8.74)	0.009965	8.0316	(0.00001, 17.19)	0.008841
*Downregulated in HGSC*						
ACTA2‐AS1	0.0804	(0.00001, 0.26)	0.02981	0.2929	(0.02, 0.57)	0.028693
ADAMTS9‐AS2	0.0483	(0.02, 0.08)	0.000044	0.0246	(0.00, 0.04)	0.003201
CBR3‐AS1	0.2967	(0.20, 0.40)	0.000222	0.3691	(0.23, 0.51)	0.001895
HAND2‐AS1	0.0206	(0.01, 0.03)	0.000118	0.0261	(0.01, 0.04)	0.000731
IPW	0.1221	(0.02, 0.23)	0.000111	0.1091	(0.01, 0.21)	0.000493
LINC00312	0.1334	(0.00001, 0.29)	0.021831	0.183	(0.06, 0.31)	0.002766
LINC00887	0.3612	(0.12, 0.61)	0.008175	0.1311	(0.00001, 0.27)	0.008167
MEG3	0.0974	(0.01, 0.18)	0.001565	0.0438	(0.01, 0.08)	0.000042
NBR2	0.212	(0.08, 0.35)	0.000705	0.1621	(0.05, 0.27)	0.00078
TSIX	0.0599	(0.01, 0.11)	0.001156	0.2033	(0.05, 0.35)	0.002829
XIST	0.2143	(0.11, 0.32)	0.000329	0.3553	(0.06, 0.65)	0.030877

**FIGURE 1:**
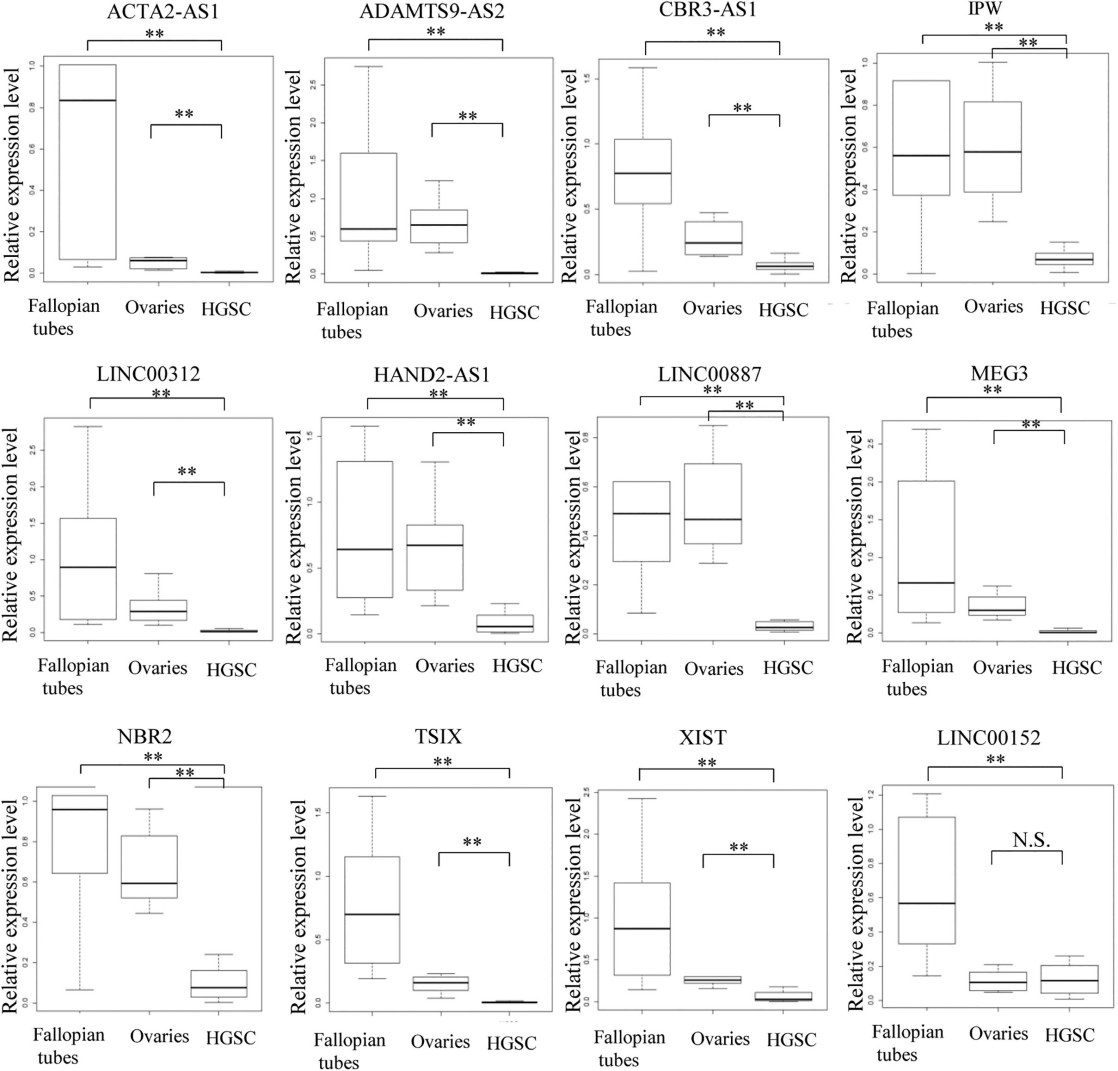
Validation of the specifically expressed lncRNAs in HGSC in multiple samples of HGSC (*n* = 22), normal fallopian tube tissue (*n* = 10) and normal ovarian tissues (*n* = 10). Box plots of relative expression levels of twelve lncRNA candidates in the twenty‐two HGSC tissue samples, ten normal fallopian tube tissue samples, and ten normal ovary tissue samples which were selected by real‐time RT‐PCR in duplicate using a PCR‐array. The relative expression levels were calculated using the mean value of normal fallopian tube tissues as one. GAPDH was used as an internal control. **p < 0.01. The expression levels in 11 lncRNA candidates (ACTA2‐AS1, ADAMTS9‐AS2, CBR3‐AS1, HAND2‐AS1, IPW, LINC00312, LINC00887, MEG3, NBR2, TSIX and XIST) which were downregulated in HGSC were confirmed to be degreased in HGSC. On the other hand, the expression levels in one lncRNA candidate (LINC00152) which was upregulated in HGSC, was not validated to be upregulated in HGSC.

The 12 candidates were then validated by real‐time RT‐PCR using multiple samples (Figure 1), and their expression levels in individual samples are shown in Figure S1. Although LINC00152 was found to be upregulated in the PCR array, validation by real‐time RT‐PCR with multiple samples revealed that its expression level was not different from that in normal ovarian tissue and was lower than that in normal fallopian tube tissue (Figure 1). Therefore, LINC00152 was not considered to be HGSC‐specific. On the other hand, 11 downregulated lncRNA candidates showed significantly lower expression levels in HGSC in comparison to normal ovarian and fallopian tube tissues in the validation experiment using multiple samples (Figure 1). Thus, 11 downregulated candidates were identified as lncRNAs specific to HGSC.

### 3.2 Establishment of HGSC cell lines overexpressing lncRNAs

**FIGURE 2:**
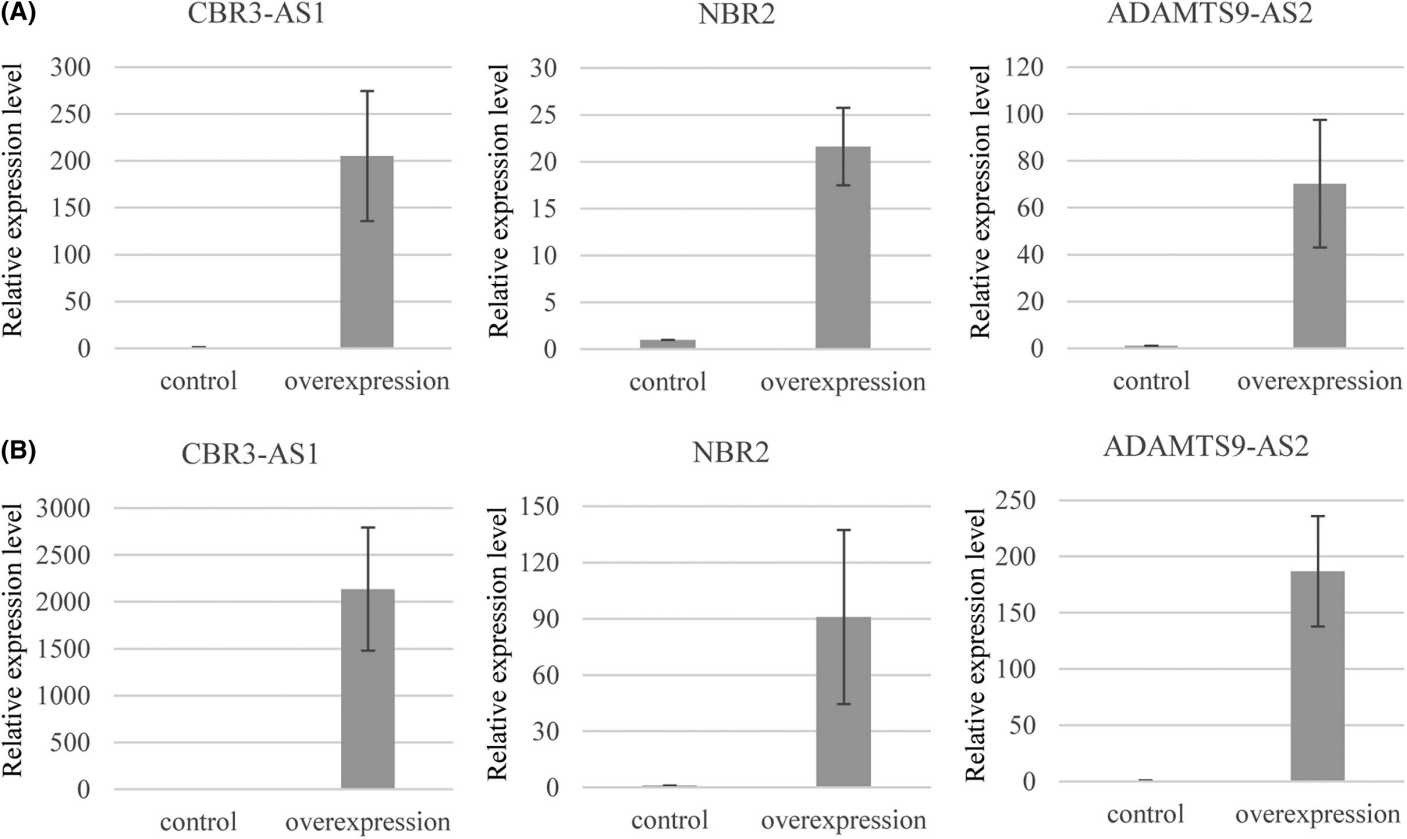
Expression levels of lncRNAs (CBR3‐AS1, NBR2 and ADAMTS9‐AS2) in the established HGSC cell lines overexpressing CBR3‐AS1, NBR2 and ADAMTS9‐AS2, respectively. Confirmation of the overexpression of CBR3‐AS1, NBR2 or ADAMTS9‐AS2 in OVCAR3 cell lines (A) and CaOV3 cell lines (B), respectively. Expression levels of CBR3‐AS1, NBR2 and ADAMTS9‐AS2 to GAPDH in each overexpressing and control cell line (empty vector) were analyzed in duplicate by real‐time RT‐PCR and are shown relative to the control cell line (as one). Values show the mean ± SD of three independent experiments (*n* = 3).

To analyze the function of the identified downregulated lncRNAs, two HGSC cell lines (CaOV3 and OVCAR3) overexpressing the lncRNAs CBR3‐AS1, NBR2, and ADAMTS9‐AS2, were established. The levels of overexpressed lncRNAs in each established cell line were confirmed by real‐time RT‐PCR (Figure 2). We also attempted to overexpress these eight lncRNAs. However, overexpressed cell lines could not be established because the full‐length lncRNAs were not amplified by real‐time RT‐PCR, or expression vectors for the lncRNAs could not be constructed.

### 3.3 Proliferation of the established cell lines

**FIGURE 3:**
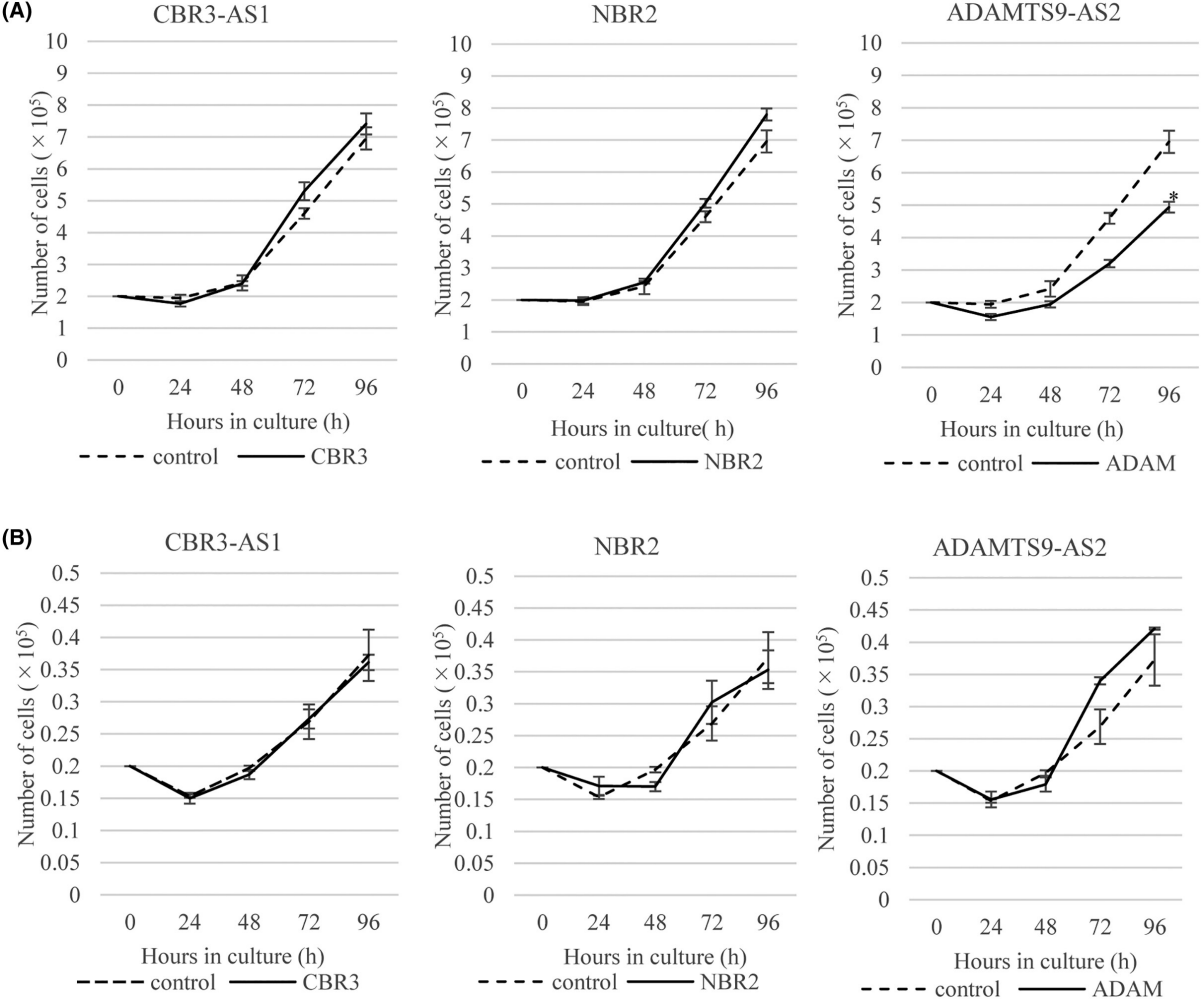
Cell proliferation assays in the established cell lines. The cell numbers in OVCAR3 cell lines (A) and CaOV3 cell lines (B) overexpressing CBR3‐AS1, NBR2 and ADAMTS9‐AS2, respectively, and control cells were counted every 24 h. Each experiment was carried out in triplicate. Values show the mean ± SD of three independent experiments (*n* = 3). *p < 0.05.

In the OVCAR3 cell lines overexpressing ADAMTS9‐AS2, cell proliferation was significantly inhibited, and in those overexpressing CBR3‐AS1 or NBR2, cell proliferation was not significantly different from that in the control cells (Figure 3A). In the CaOV3 cell lines overexpressing CBR3‐AS1, NBR2, or ADAMTS9‐AS2, cell proliferation was not significantly different from that of the control cells (Figure 3B).

### 3.4 Wound healing assay of the established cell lines

**FIGURE 4:**
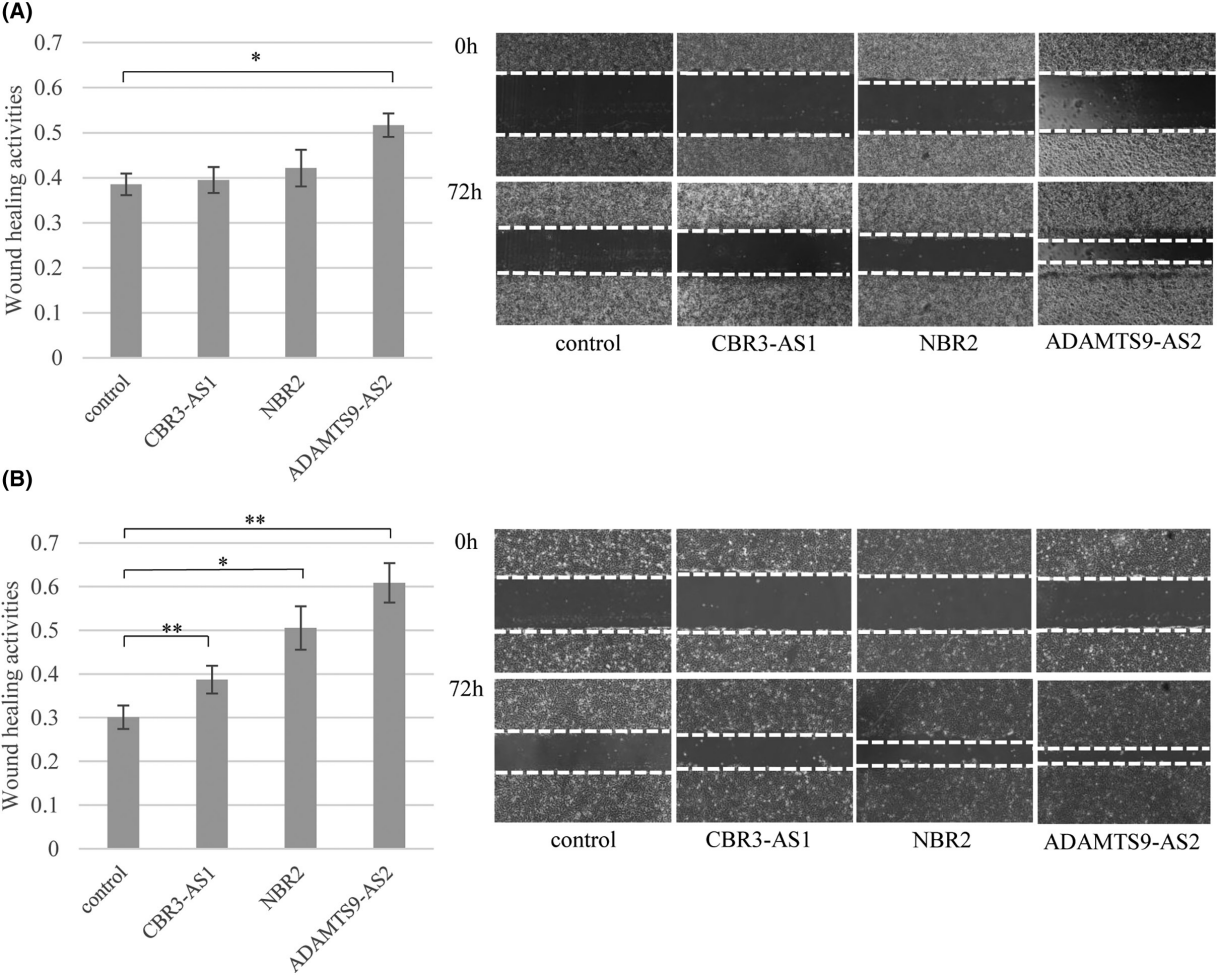
Wound healing assays in the established cell lines. Wound healing assay in the cells overexpressing CBR3‐AS1, NBR2 or ADAMTS9‐AS2, respectively, and control cells in OVCAR3 cell lines (A) and CaOV3 cell lines (B). Images were obtained at the same fields, 0 and 72 h after wound creation (right panels). Horizontal dotted lines indicate the wound margins. Wound healing activities (left panels) were calculated by dividing the migration distance at 72 h by the original distance at 0 h. Each experiment was carried out in triplicate. Values indicate the mean ± SD of three independent experiments (*n* = 3). *p < 0.05 and **p < 0.01 in comparison to control.

In OVCAR3 cell lines overexpressing ADAMTS9‐AS2, the wound healing activity was significantly higher than that in control cells (Figure 4A). There was no difference in wound healing activities between cells overexpressing CBR3‐AS1 or NBR2 and control cells (Figure 4A). In CaOV3 cell line overexpressing CBR3‐AS1, NBR2, or ADAMTS9‐AS2, the wound healing activity was higher than that in control cells (Figure 4B).

### 3.5 Activities of cell migration and cell invasion of the established cell lines

**FIGURE 5:**
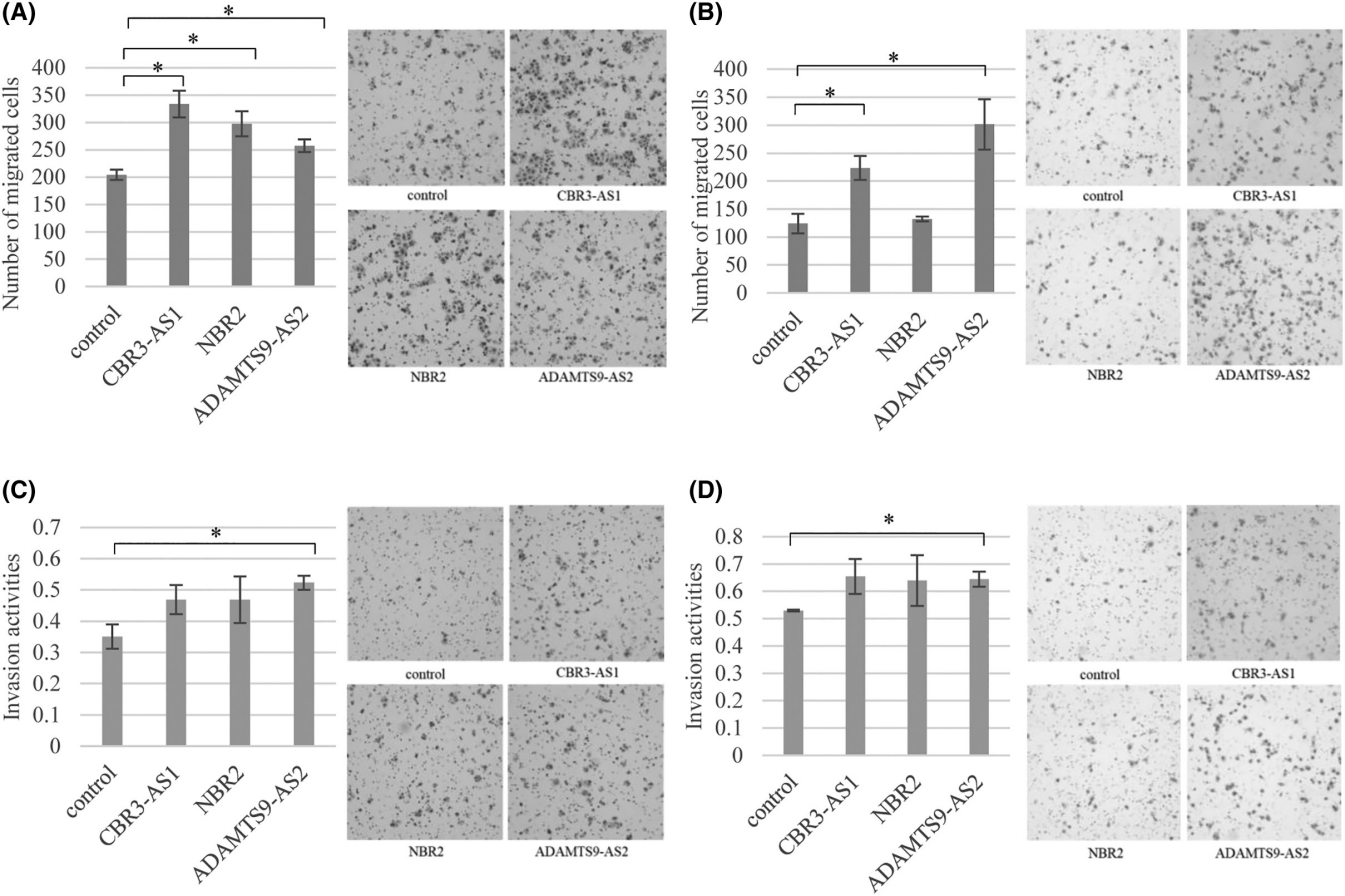
Cell migration and cell invasion assays in the established cell lines. Cell migration assays in the cells overexpressing CBR3‐AS1, NBR2 or ADAMTS9‐AS2, respectively, and control cells in OVCAR3 cell lines (A) and CaOV3 cell lines (B). Representative photographs of the migrated cells stained by Diff‐Quick are shown. The numbers of the migrated cells were counted in five randomized fields and the mean was calculated. Each experiment was carried out in triplicate. Values represent the mean ± SD of three independent experiments (*n* = 3). *p < 0.05 in comparison to control. Cell invasion assay in the cells overexpressing CBR3‐AS1, NBR2 or ADAMTS9‐AS2 and control cells in OVCAR3 cell lines (C) and CaOV3 cell lines (D). Representative photographs of the invaded cells stained by Diff‐Quick. The invasion activities were calculated by the numbers on the Matrigel‐coated membrane divided by the number of cells on the uncoated membrane. Each experiment was carried out in triplicate. Values represent the mean ± SD of three independent experiments (*n* = 3). *p < 0.05 in comparison to control.

In the OVCAR3 cell lines overexpressing CBR3‐AS1, NBR2, and ADAMTS9‐AS2, the number of cells that migrated across the membranes was significantly higher than that observed in the control cells (Figure 5A). In the CaOV3 cell lines overexpressing CBR3‐AS1 and ADAMTS9‐AS2 (Figure 5B), the number of migrated cells was also higher than that in the control cells. However, in the cell line overexpressing NBR2, the number of migrated cells did not differ from that in the control cells.

Invasion activity was evaluated by dividing the number of cells that migrated through the Matrigel‐coated membranes by the number of cells that migrated through the uncoated membranes. In the OVCAR3 cell line overexpressing ADAMTS9‐AS2, the invasion activity was significantly higher than that in the control cells, whereas there was no difference in invasion activity between the cells overexpressing CBR3‐AS1 or NBR2 and the control cells (Figure 5C). Similarly, in the CaOV3 cell line overexpressing ADAMTS9‐AS2, the invasion activity was significantly increased in comparison to the control cells and was not different from that in the cells overexpressing CBR3‐AS1 or NBR2 (Figure 5D).

## 4 DISCUSSION

In this study, 11 HGSC‐specific lncRNAs that were downregulated in HGSC were identified by comparing HGSC tissue samples with normal ovarian and fallopian tube tissue samples. We previously identified four lncRNAs specific to HGSC based on the screening of ovarian cancer cell lines and primary ovarian surface epithelial cells.^12^ Three of the lncRNAs (ADAMTS9‐AS2, MEG3, and XIST) were consistent with those identified in this study. In addition, MON2‐AS1, LINC00399, and AC002115.1, which were identified by Nicholas et al.^11^ as lncRNAs involved in survival of patients with HGSC, did not match the lncRNAs identified in this study. Eight new lncRNAs specific to HGSC (ACTA2‐AS1, CBR3‐AS1, HAND2‐AS1, IPW, LINC00312, LINC00887, NBR2, and TSIX) were identified in the present study.

Eight of the 11 lncRNAs identified in this study have been reported to have expression profiles or functional roles in ovarian cancer. XIST, MEG3, IPW, HAND2‐AS, ADAMTS9‐AS2, and LINC00312 have been reported to be downregulated in ovarian cancers in several studies,^15^, ^16^, ^17^, ^18^, ^19^, ^20^ which is consistent with the present study. XIST and MEG3 have been reported to suppress cell migration and invasion in ovarian cancer.^15^, ^16^ HAND2‐AS1 was reported to act as a tumor suppressor in HGSC cell lines.^18^ ADAMTS9‐AS2 inhibits cell proliferation, invasion, and epithelial‐mesenchymal transition.^19^ LINC00312 inhibits cell proliferation and promotes apoptosis. In addition, the expression of LINC00312 in chemoresistant serous epithelial ovarian carcinoma tissues is significantly lower than that in chemosensitive tissues.^20^ In contrast to the expression of lncRNAs in HGSCs in this study, the expression of ACTA2‐AS1 and NBR2 has been reported to be upregulated in ovarian cancers.^21^, ^22^, ^23^ ACTA2‐AS1 has been reported to be associated with cell proliferation and invasion in ovarian cancers^21^ and is associated with cisplatin resistance in ovarian cancer cell lines and tissues.^22^ As for NBR2, patients that expressed it more strongly tended to have a better prognosis.^23^ However, there are no reports of ovarian cancers expressing CBR3‐AS1, LINC00887, and TSIX; this is the first report of their expression in ovarian cancers, including HGSC.

In this study, overexpression cell lines were established for three of the 11 identified lncRNAs (CBR3‐AS1, NBR2, and ADAMTS9‐AS2) to conduct a functional analysis. The functional analyses are summarized in Table 3, and previous reports related to various cancers involving each lncRNA are listed in Table 4. CBR3‐AS1 (also known as PlncRNA‐1) was first found to be upregulated in prostate cancer cell lines and tissues.^24^ Previous studies have shown that CBR3‐AS1 is upregulated in several cancers, including breast cancer,^25^ colorectal cancer,^26^ and osteosarcoma.^27^ Furthermore, CBR3‐AS1 has been reported to be associated with a poor prognosis and the promotion of malignant behaviors in these cancers (Table 4).^25^, ^26^, ^27^ However, in this HGSC study, unlike in studies of many other cancer types, CBR3‐AS1 was downregulated, whereas its overexpression promoted migration activity in vitro.

**TABLE 3:** The summary of functional analyses in this study.

	CBR3‐AS1		NBR2		ADAMTS9‐AS2	
	OVCAR3	CaOV3	OVCAR3	CaOV3	OVCAR3	CaOV3
Proliferation assay	n.s.	n.s.	n.s.	n.s.	Suppression	n.s.
Wound healing assay	n.s.	Promotion	n.s.	Promotion	Promotion	Promotion
Migration assay	Promotion	Promotion	Promotion	n.s.	Promotion	Promotion
Invasion assay	n.s.	n.s.	n.s.	n.s.	Promotion	Promotion

**TABLE 4:** Previous reports related with three lncRNAs (CBR3‐AS1, NBR2, and ADAMTS9‐AS2) in various cancers.

	CBR3‐AS1		NBR2		ADAMTS9‐AS2	
	Expression level & functions	References	Expression level & functions	References	Expression level & functions	References
Breast cancer	**Up‐regulated** poor prognosis, cell proliferation(+), apoptosis(−)	Xu L, et al.^25^				
Clear cell renal cell carcinoma					**Down‐regulated**	Song E, et al.^44^
Colorectal cancer	**Up‐regulated** poor prognosis cell proliferation(+), migration(+), invasion(+)	Xie L, et al.^26^	**Down‐regulated** proliferation(−) migration(−)	Lai F, et al.^59^	**Down‐regulated** cell proliferation(−), migration(−), invasion(−)	Liu W, et al.^61^
	**Down‐regulated** migration(+), invasion(+)	Ye W, et al.^36^				
Endometrial cancer			**Down‐regulated**	Dong P, et al.^60^		
Esophageal cancer					**Down‐regurated** cell proliferation(−), migration(−), invasion(−)	Chen Z, et al.^31^
Gastric cancer					**Down‐regulated** cell proliferation(−), migration(−) cisplatin sensitivity(+)	Ren N, et al.^32^
Glioma			**Up‐regulated** poor prognosis, cell proliferation(+), migration(+), invasion(+)	Zhang J, et al.^37^		
Hepatoblastoma			**Up‐regulated** cell proliferation(+), migration(+), invasion(+)	Zhu C, et al.^38^		
Liver cancer					Cell proliferation(−), migration(−), invasion(−)	Li H, et al.^33^
Lung adenocarcinoma					**Down‐regulated**	Lin Z, et al.^34^
Non‐small cell lung cancer	**Up‐regulated** poor prognosis, cell proliferation(+), migration(+), invasion(+)	Guany, et al.^58^	**Down‐regulated** cell proliferation(−), migration(−), invasion(−)	Ygao YP, et al.^28^	**Down‐regulated**	Sulewska A, et al.^62^
Osteosarcoma	**Up‐regulated** poor prognosis, cell proliferation(+), migration(+), invasion(+), apoptosis(−)	Zhang Y, et al.^27^	**Down‐regulated** cell proliferation(−), migration(−), invasion(−)	Cai W, et al.^29^		
Prostate cancer	**Up‐regulated** cell proliferation(+), apoptosis(−)	Cui Z, et al.^24^			**Down‐regulated**	He L, et al.^35^
Thyroid cancer			**Down‐regulated** cell proliferation(−), migration(−), invasion(−), apoptosis(+)	Yang W, et al.^30^		

Abbreviations: (+), promoted; (−), inhibit.

NBR2 (the neighbor of BRCA1 gene 2) is downregulated in various cancers and acts as a tumor suppressor (Table 4).^28^, ^29^, ^30^ In this study, although NBR2 was downregulated in HGSC tissues, which is consistent with its behavior in other cancer types, its overexpression promoted cell migration in vitro.

ADAMTS9‐AS2 inhibited cell proliferation, migration, and invasion in several cancers and was downregulated in esophageal, gastric, and liver cancers (Table 4).^31^, ^32^, ^33^ In addition, the downregulation of ADAMTS9‐AS2 has been correlated with a poor prognosis in lung adenocarcinoma and prostate cancer (Table 4).^34^, ^35^ In this study, ADAMTS9‐AS2 was downregulated in HGSC tissue, and its overexpression suppressed cell proliferation, which is consistent with its behavior in other cancer types, while its overexpression promoted cell migration and invasion activities in vitro.

For two lncRNAs (CBR3‐AS1 and NBR2), there seemed to be discrepancies between their expression levels in HGSC tissues and their malignant behaviors in vitro (Table 4). This is consistent with other studies showing that these lncRNAs promote some cancer types and inhibit others. Although CBR3‐AS1 is often reported to be upregulated in various cancer types,^24^, ^25^, ^26^, ^27^ it has been reported that CBR3‐AS1 is downregulated in colorectal cancer, and its overexpression induces cell migration and invasion by absorbing miR‐29a in vitro.^36^ Furthermore, although NBR2 is downregulated in most cancers, it is upregulated and promotes cell proliferation, migration, and invasion in glioma and hepatoblastoma.^37^, ^38^ Since lncRNAs have multiple functions, including transcriptional regulation, nuclear domain organization, and regulation of proteins and RNA molecules,^39^ their functions may differ among different cancer types. In addition, since lncRNAs usually interact with other factors such as microRNAs, mRNAs, and proteins,^36^, ^38^, ^40^ the differences in the interactions between these molecules in each cancer type may alter their ability to affect malignant behaviors.

ADAMTS9‐AS2 is downregulated and inhibits cell proliferation, migration, and invasion in most cancers (Table 4). However, in this study, overexpression of ADAMTS9‐AS2 suppressed cell proliferation in the OVCAR3 cell line, whereas it promoted cell migration and invasion in both cell lines. There is no clear explanation for these contradictory cell behaviors. The association between ADAMTS9‐AS2 and TGFβ or FOXO1 may help to explain the opposite phenomenon that was observed in this study. ADAMTS9‐AS2 controls cancer cell behavior by modulating TGF‐β signaling in breast cancer and tongue squamous cell carcinoma.^41^, ^42^ Interestingly, TGFβ is reported to promote cell migration and metastasis by inducing epithelial‐mesenchymal transition (EMT), while inhibiting cell proliferation by inducing G1 phase cell cycle arrest in human oral cancer cells.^43^ In addition, ADAMTS9‐AS2 regulates FOXO1 in clear cell RCC.^44^ FOXA1, a gene family that includes FOXO1, promotes cell proliferation and inhibits cell motility and EMT in prostate cancer cells.^45^ These findings raise the possibility that ADAMTS9‐AS2 is involved in contradictory cell behaviors by regulating factors such as TGF‐β and FOXO1, which potentially have dual effects on cancer cell behavior. However, because lncRNAs have various functions, further studies are needed to explain the contradictory cell behaviors induced by the overexpression of ADAMTS9‐AS2.

There may an alternative possibility for contradictory cell behaviors observed in this study. The expression levels of ADAMTS9‐AS2, CBR3‐AS1, and NBR2 in the overexpression experiment using cell lines are higher than those in HGSC tissues compared with fallopian tubes and ovaries. As described above, there were several contradictory cell behaviors induced by the overexpression of ADAMTS9‐AS2, CBR3‐AS1, and NBR2. We cannot neglect the possibility that remarkable overexpression may have affected the cellular behaviors of cell lines.

It is interesting to note that normal fallopian tube and ovary tissues strongly expressed the lncRNAs identified in this study (Figure S1). The function of these lncRNAs in the fallopian tubes and ovaries is unclear although other lncRNAs have been reported to be involved in reproduction.^46^ The lncRNAs identified in this study may play a role in the physiological functions through the second messengers. Actions of ADAMTS9‐AS2 have been reported to be mediated by TGFβ.^41^, ^42^ TGFβ is expressed in the fallopian tube and ovary, and may contribute to development of the early embryo for implantation and follicle development, respectively.^47^, ^48^, ^49^ CBR3‐AS1 and NBR2 work through Notch signaling,^29^, ^50^ which is involved in cellular remodeling in the fallopian tube and granulosa cell differentiation and ovarian steroid hormone production in the ovary.^51^ NBR2 also interacts with AMP‐activated protein kinase (AMPK).^52^ AMPK is reported to be involved in autophagy of granulosa cells and oocytes, which regulates follicle development,^53^ and AMPK in the fallopian tube may contribute to embryonic development, spermatocyte DNA damage repair, and ovarian steroid hormone production.^54^

Furthermore, the expression of ADAMTS9‐AS2, CBR3‐AS1, and NBR2 in the fallopian tube tends to be lower in young women. Although the reason for the age‐dependent expression is unclear, these lncRNAs may be repressed by estrogen because it is reported that some lncRNAs are increased and some are repressed by estrogen.^55^

In addition, the expression levels of lncRNAs identified in this study seem to be higher in the fallopian tube than in the ovary. However, we have no convincing answer to explain this finding. Ovaries are consisted of many kinds of cells including ovarian surface epithelium, stromal cells, and fibroblasts, etc. while fallopian tubes are mainly consisted of epithelial cells. Although the localization of these lncRNAs in the fallopian tube and ovary is unclear, the different expression levels of these lncRNAs may be due to the difference in cellular component between the fallopian tube and ovary.

The present study was associated with some limitations. P53 is known to be involved in the pathogenesis of HGSC.^56^ The expression of p53 and p53 driver mutation was not examined in HGSC samples, normal ovary tissues, or normal fallopian tube tissues. Since p53 has been reported to regulate lncRNAs,^40^, ^57^ the presence or absence of p53 driver mutations may influence the expression of lncRNAs. In addition, the patients' age at the time of sampling of normal ovary and fallopian tube tissues varied among the patients. The possibility that the expression of lncRNAs is influenced by age cannot be neglected.

In conclusion, we identified 11 downregulated lncRNAs in HGSC patients. Three of these, CBR3‐AS1, NBR2, and ADAMTS9‐AS2, were found to be involved in the malignant behavior of HGSC. These lncRNAs do not necessarily suppress malignant behaviors of HGSC. Further studies are needed to clarify the detailed mechanism by which these lncRNAs regulate the malignant behavior of HGSC.

## FUNDING INFORMATION

This work was supported in part by JSPS KAKENHI Grants 23K08889, 23K08824, 23K08870, 23K07312, 23K15838, 22K09620, 21K16816, 21K09517, 21K09495, and 21K09542 for Scientific Research from the Ministry of Education, Science, and Culture, Japan.

## CONFLICT OF INTEREST STATEMENT

The authors declare that there are no conflicts of interest. Norihiro Sugino is an Editorial Board member of Reproductive Medicine and Biology and a co‐author of this article. To minimize bias, he was excluded from all editorial decision‐making related to the acceptance of this article for publication.

## HUMAN RIGHTS STATEMENTS AND INFORMED CONSENT

All experiments involving human tissues were conducted in accordance with the Declaration of Helsinki. Informed consent was obtained from all the patients before sample collection.

## APPROVAL BY ETHICS COMMITTEE

All experiments involving human tissues were conducted in the protocol was approved by the Institutional Review Board of Yamaguchi University Graduate School of Medicine (No. H27‐216).

## Supporting information


Figure S1

Table S1

Table S2

Table S3
