# Supplementary figures and images for: Identification of long noncoding RNAs downregulated specifically in ovarian high‐grade serous carcinoma
Source: Reprod Med Biol. 2024 Apr 3;23(1):e12572. doi: 10.1002/rmb2.12572 (PMC10988898; doi:10.1002/rmb2.12572)

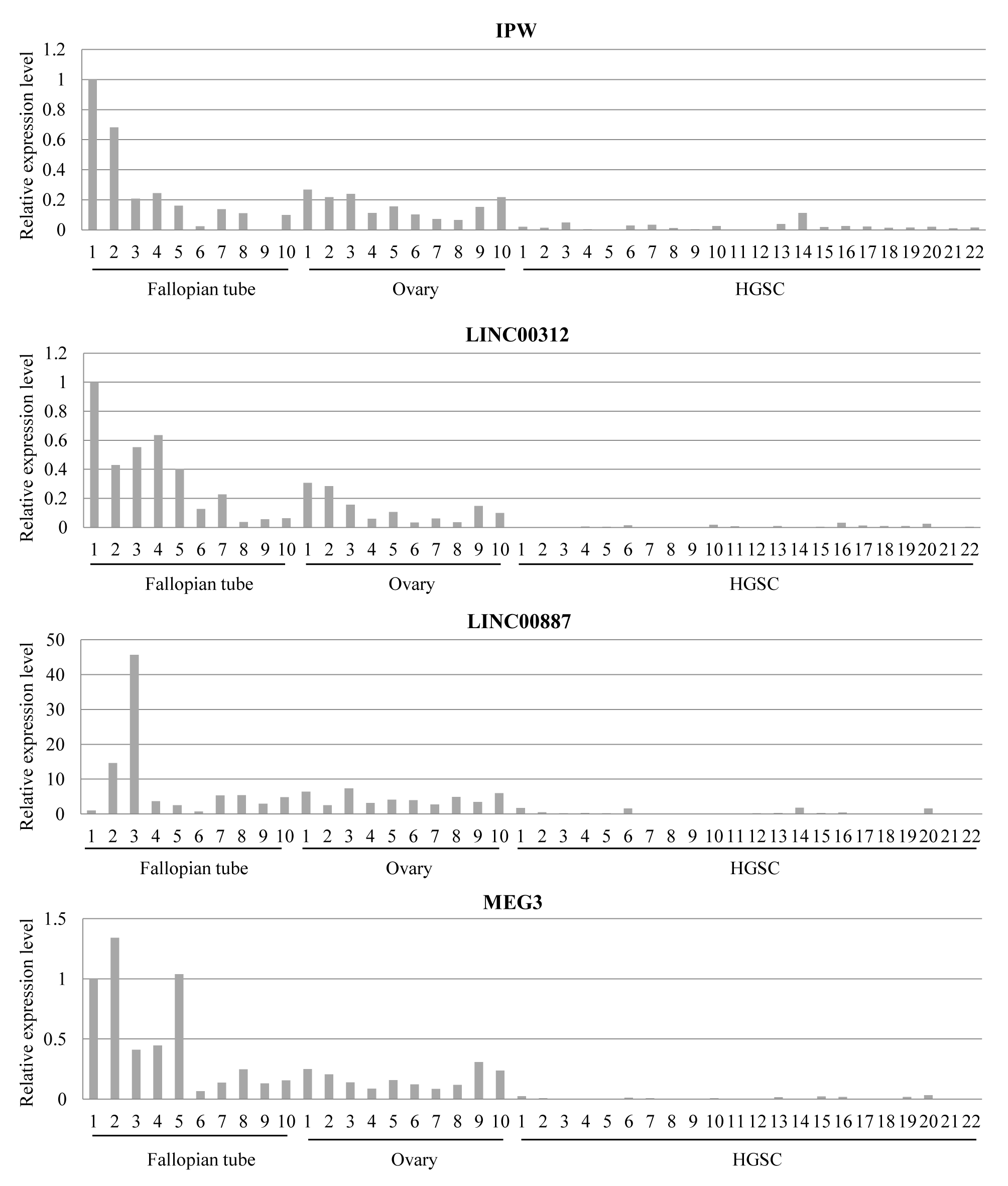

Supplement: Supplementary file 1 — Figure S1 [file RMB2-23-e12572-s001.zip › FigureS1 continuance1.tif]

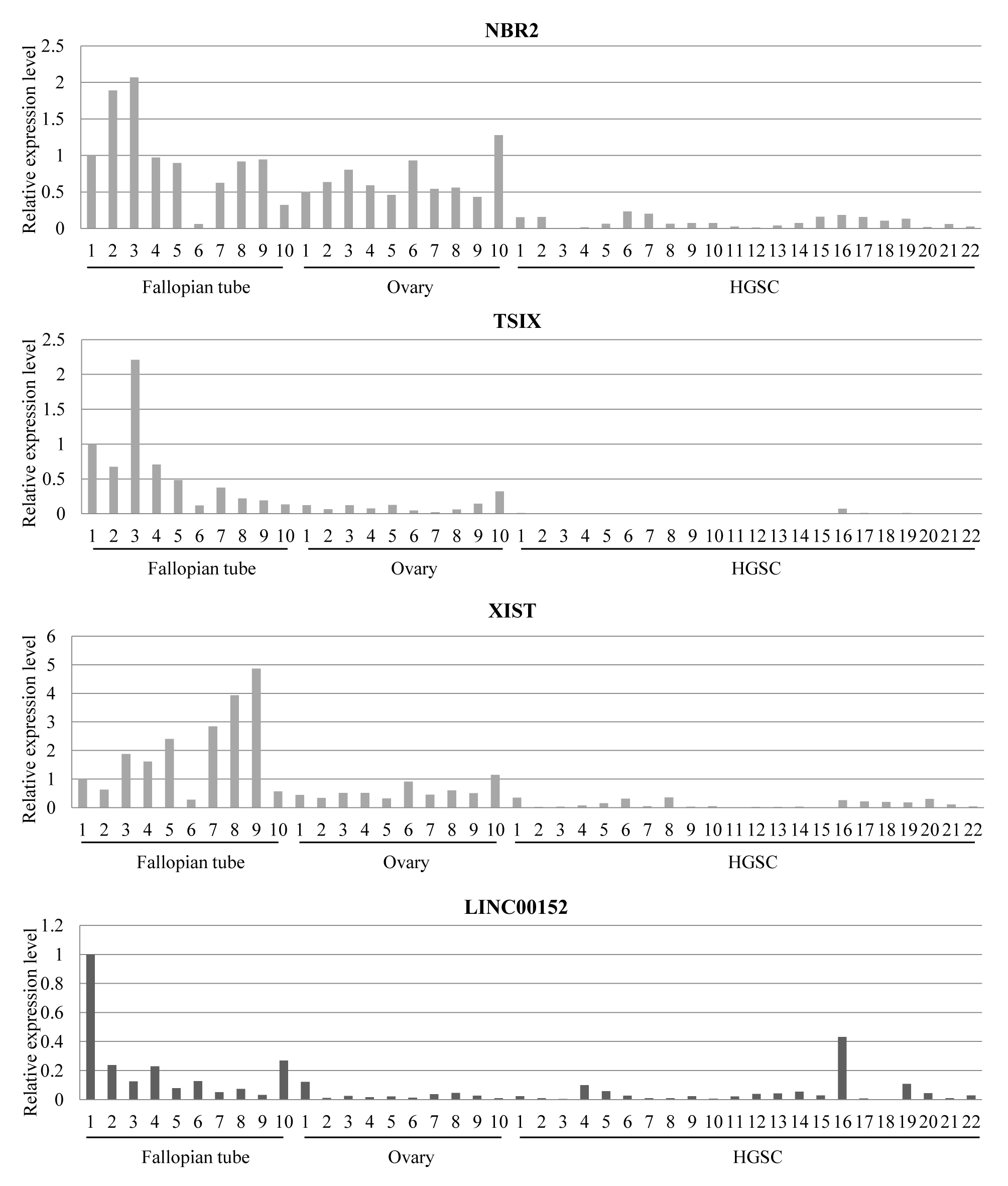

Supplement: Supplementary file 1 — Figure S1 [file RMB2-23-e12572-s001.zip › FigureS1 continuance2.tif]

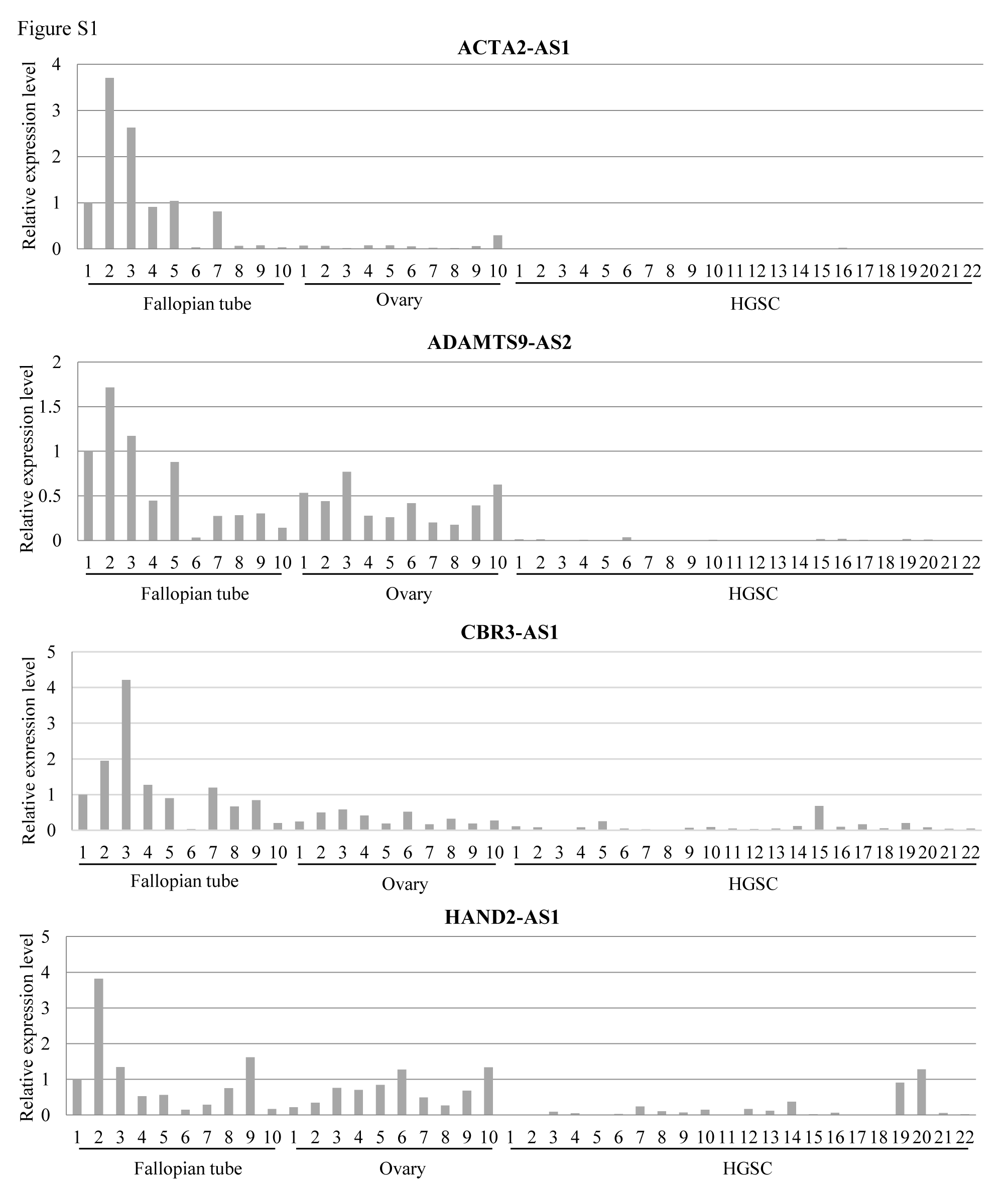

Supplement: Supplementary file 1 — Figure S1 [file RMB2-23-e12572-s001.zip › FigureS1.tif]
