# Supplementary material for: Identification of long noncoding RNAs downregulated specifically in ovarian high‐grade serous carcinoma
Source: Reprod Med Biol. 2024 Apr 3;23(1):e12572. doi: 10.1002/rmb2.12572 (PMC10988898; doi:10.1002/rmb2.12572)
Supplement: Supplementary file 2 — Table S1 [file RMB2-23-e12572-s003.docx]

Table S1 Clinical data from twenty-two HGSC cases

| No | Age | FIGO stage | CA125  at diagnosis | CA19-9  at diagnosis | CEA  at diagnosis | Residual disease |
| --- | --- | --- | --- | --- | --- | --- |
| 1 | 62 | IC2 | 37 | 14 | 1.7 | complete |
| 2 | 84 | IIIB | 1434 | 1 | unknown | suboptimal |
| 3 | 61 | IIIB | 824 | 6 | 2 | optimal |
| 4 | 56 | IIIC | 548 | 2 | <0.5 | suboptimal |
| 5 | 45 | IIIC | 696 | 10 | unknown | suboptimal |
| 6 | 51 | IIIC | 920 | 1 | 1.4 | suboptimal |
| 7 | 63 | IIIC | 376 | 14 | 1.8 | suboptimal |
| 8 | 74 | IIIC | 7044 | 18 | unknown | suboptimal |
| 9 | 74 | IIIC | 850 | <1 | 15.8 | complete |
| 10 | 72 | IIIC | 3230 | 19 | 2.5 | suboptimal |
| 11 | 74 | IIIC | 1201 | 18 | 5.2 | suboptimal |
| 12 | 65 | IVA | 3821 | 7 | 2 | suboptimal |
| 13 | 53 | IVA | 921 | 16 | 2.9 | optimal |
| 14 | 81 | IVB | 127 | 20 | 1.8 | optimal |
| 15 | 64 | IVB | 853 | 20 | unknown | suboptimal |
| 16 | 64 | IVB | 1914 | 12 | 0.7 | suboptimal |
| 17 | 80 | IVB | 41 | 18 | unknown | optimal |
| 18 | 54 | IVB | 4772 | 1 | 31 | optimal |
| 19 | 77 | IVB | 5455 | 33 | unknown | suboptimal |
| 20 | 74 | IVB | 4616 | <0.5 | 2 | suboptimal |
| 21 | 40 | IVB | 1245 | unknown | unknown | suboptimal |
| 22 | 77 | IVB | 975 | 1 | 2.7 | suboptimal |
