# Supplementary material for: Identification of long noncoding RNAs downregulated specifically in ovarian high‐grade serous carcinoma
Source: Reprod Med Biol. 2024 Apr 3;23(1):e12572. doi: 10.1002/rmb2.12572 (PMC10988898; doi:10.1002/rmb2.12572)
Supplement: Supplementary file 4 — Table S3 [file RMB2-23-e12572-s004.docx]

Table S3 Clinical data on the patients whom normal falloian tubes were obtained from

| No | Age | Clininical diagnosis | Pathological diagnosis | Fallopian tubes used in this study (Rt or Lt) |
| --- | --- | --- | --- | --- |
| 1 | 68 | Endomertial cancer | endometrioid carcinoma, Grade 2 | Rt |
| 2 | 66 | Uterine fibroid | Uterine leiomyoma | Rt |
| 3 | 59 | Lt. ovarian cyst | mucinous cystadenoma | Rt |
| 4 | 71 | Lt. ovarian cancer | clear cell carcinoma | Rt |
| 5 | 68 | Lt. ovarian cancer | high grade neuroendocrine carcinoma | Rt |
| 6 | 40 | Cervical cancer | Squamous cell carcinoma | Rt |
| 7 | 53 | Uterine fibroid | Uterine leiomyoma | Rt |
| 8 | 57 | Endomertial cancer | endometrioid carcinoma, Grade 1 | Rt |
| 9 | 44 | Uterine fibroid | Uterine leiomyoma | Rt |
| 10 | 42 | Uterine fibroid | Uterine leiomyoma | Rt |
